# Detection of *Rickettsia* spp. in questing ticks (Acari: Ixodidae) from forest fragments adjacent to agricultural and livestock farms in Casanare, Colombia

**DOI:** 10.1007/s00436-025-08484-2

**Published:** 2025-04-24

**Authors:** José Luis Rodríguez-Bautista, Patricia Fuya-Oviedo, Ard Menzo Nijhof, Lidia Chitimia-Dobler, Isaiah Obara, Adivaldo Fonseca

**Affiliations:** 1 Facultad de Medicina Veterinaria y de Zootecnia, Universidad Nacional de Colombia, Bogotá, Colombia; 2 Laboratorio de Entomología, Instituto Nacional de Salud de Colombia, Bogotá, Colombia; 3 Institute for Parasitology and Tropical Veterinary Medicine, Freie Universität Berlin, Berlin, Germany; 4 Veterinary Centre for Resistance Research, Freie Universität Berlin, Berlin, Germany; 5 Bundeswehr Institute of Microbiology, Munich, Germany; 6 Fraunhofer Institute of Immunology, Infection and Pandemic Research, Penzberg, Germany; 7 Programa de Pós-Graduação em Ciências Veterinárias, Universidade Federal Rural do Rio de Janeiro, UFRRJ, Seropédica, RJ Brazil

**Keywords:** *Amblyomma*, Spotted fever group *Rickettsia*, Tick-borne pathogens

## Abstract

This study aimed to describe the diversity of ixodid tick species in rural forest areas of Yopal and Aguazul, Department of Casanare, Colombia, and to evaluate the presence of tick-borne *Rickettsia* sp., potentially impacting animal and human health. A cross-sectional study was conducted on questing ticks collected from forested farm areas that keep livestock and/or conduct agricultural activities. All ticks were subjected to DNA extraction and tested by PCR amplifying fragments of three rickettsial genes: *gltA*, *htrA*, and *ompA*. A total of 852 individual ticks (larvae, nymphs, and adults) and 15 additional larvae clusters were collected and identified as *Amblyomma mixtum*, *Amblyomma* cf*. parvum*, *Amblyomma dissimile*, *Amblyomma* spp., and *Dermacentor* spp. *Rickettsia* organisms were detected by PCR in 7.0% (16/230) of the individual larvae, 0.2% (1/488) of the nymphs, and 1.5% (2/134) of the adults. “*Candidatus* Rickettsia colombianensi” and *Rickettsia amblyommatis* were detected in 1.52% (13/852) and 0.47% (4/852) of the ticks, respectively. In addition, a novel genotype, *Rickettsia* sp. genotype Yopal, phylogenetically closest to *Rickettsia canadensis,* was identified in two *A. mixtum* males. Several tick and *Rickettsia* species were found in the study areas, suggesting the need for an in-depth study of nonspecific acute tropical febrile illness in the Department of Casanare and across Colombia. Priority areas for future studies include investigating the molecular, clinical, and epidemiological features of the presentation of tick species and the possible participation of “*Ca*. R. colombianensi,” *R. amblyommatis*, and “*Rickettsia* sp. Yopal genotype of *A. mixtum*” to understand their role in human rickettsiosis in the study region.

**Supplementary Information:**

The online version contains supplementary material available at 10.1007/s00436-025-08484-2.

## Introduction

Hard ticks (Acari: Ixodidae) can transmit a variety of pathogens to animals and humans worldwide. Among the transmitted pathogens, bacterial agents of the spotted fever group (SFG) rickettsiae are considered of global importance due to an increasing number of cases in human populations and the wide distribution of wild and domestic animals that act as reservoirs. The human-animal interface occurs mainly in rural areas where human and animal populations frequently interact (Donalisio et al. 2020). In Colombia, the presence of clinical rickettsiosis in humans caused by SFG rickettsiae like *Rickettsia rickettsii* and *Rickettsia parkeri* strain Atlantic rainforest has been demonstrated (Acosta et al. 2006; Arboleda et al. 2020; Ávila-Aguirre et al. 2020; Cuéllar-Sáenz et al. 2023; Faccini-Martínez et al. 2016; Gómez-Quintero et al. 2017; Hidalgo 2010; M. Hidalgo et al. 2007; Luis Patiño Camargo 2006; Pacheco 2008; Patiño et al. 2006; Quintero Vélez et al. 2012). In addition, tick-associated rickettsial pathogens have been detected in ticks from Colombia (Arroyave et al. 2020; Cardona et al. 2020; Cotes-Perdomo et al. 2020; Faccini-Martínez et al. 2015; Londoño et al. 2014; Martínez-Sánchez et al. 2021; Miranda et al. 2012; Rivera-Páez et al. 2018; Sánchez et al. 2019; Santodomingo et al. 2018). Serological evidence of antibodies against rickettsial pathogens has also been found in humans and animals (Arroyave et al. 2020; Betancourt et al. 2020; Faccini-Martínez et al. 2016; Gómez-Quintero et al. 2017; Hidalgo 2010; Hidalgo et al. 2007; Ortiz et al. 2015; Quintana 2018; Quintero Vélez et al. 2012; Riveros-Pinilla et al. 2015).

The ecological and climatic suitability of the Department of Casanare for the development and expansion of tick populations has been reported (Reina-Jiménez & Tovar-Muñoz 2007). Located in the central-eastern part of Colombia in the Orinoquia region, this area has neotropical characteristics with optimal climatic and geographical conditions and protected or semi-preserved forested areas rich in fauna and flora that favor the presence and proliferation of arthropod vectors of important human and animal pathogens (Sánchez et al. 2005). The rich biodiversity and important anthropogenic activity, with the destruction of forest reserves due to the expansion of the agricultural frontier and agricultural practices themselves, make this region a high-risk area for the spread or appearance of zoonotic diseases such as rickettsiosis (Dobson & Foufopoulos 2001).

In Casanare, ticks are considered a major constraint to livestock production due to their impact on animal health. Several tick-borne diseases affecting cattle, horses, and dogs are considered endemic to the region, and suspected cases of spotted fever (SF) in humans have been reported. In the last decade, seropositivity for *Rickettsia* spp. has been documented in horses (Riveros-Pinilla et al. 2015), wild mice, and capybaras (Quintero et al 2013; Quintana 2018).

Consequently, local health authorities are concerned about cases of acute febrile illness in the human population that could correspond to SF, along with other diseases of medical and veterinary significance.

In this context, the present study aimed to describe the local tick species and investigate the presence of *Rickettsia* species in questing ticks in forest reserves adjacent to agricultural and cattle farms in the rural municipalities of Yopal and Aguazul in the Department of Casanare, Colombia.

## Materials and methods

### Ethical approval

This work was carried out with the permission of the Colombia’s National Environmental License Authority (ANLA; License No. 01300, 4 July 2019).

### Study area

**Fig. 1:**
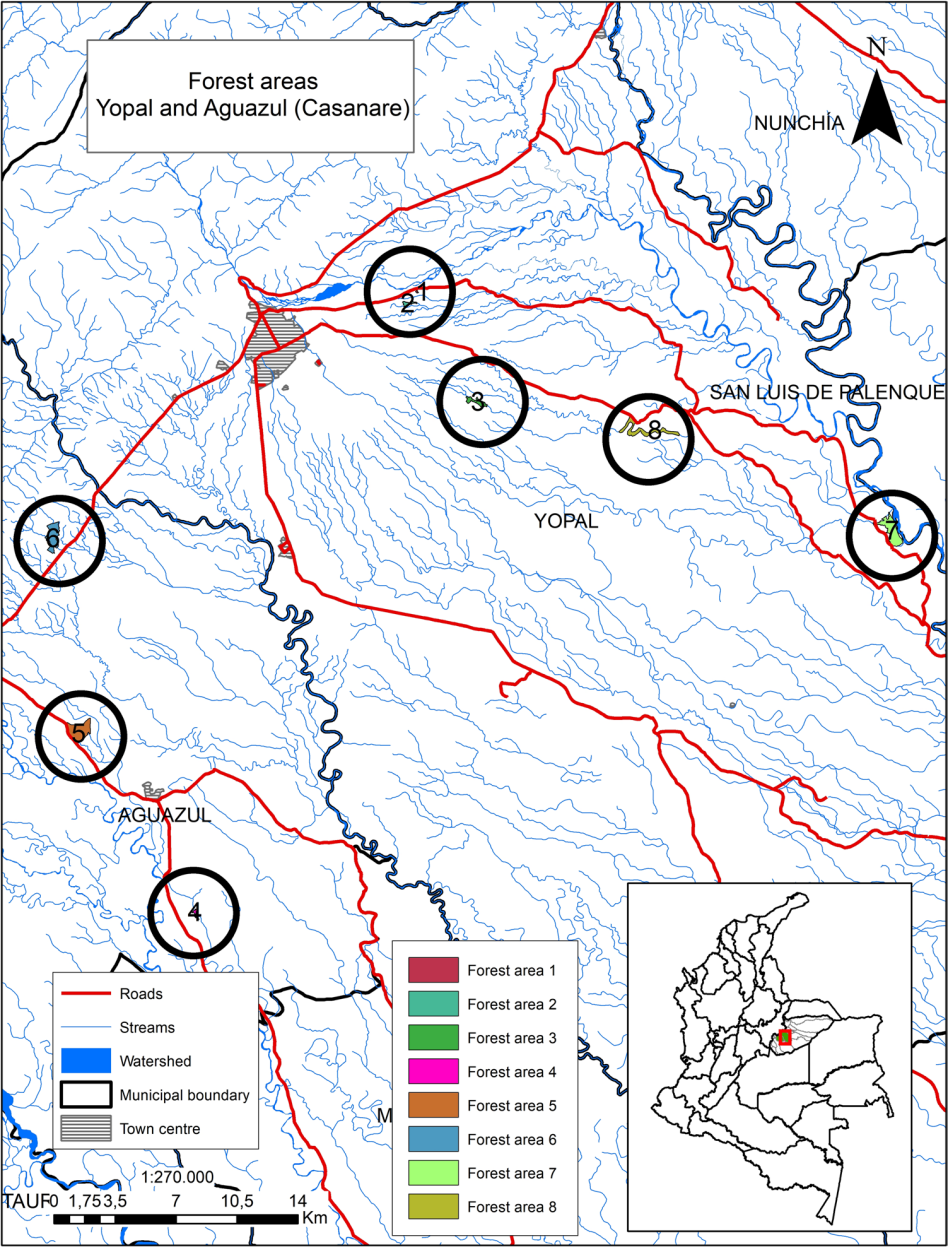
Distribution map of the eight tick collection groups in the rural area of the municipalities of Yopal and Aguazul, Department of Casanare, Colombia. Numbers in circles correspond to collecting fragments. Some of these forests are part of the protection system of water sources used for irrigation of pastures or agricultural crops in the region

Ticks were collected in March 2021 in rural areas of the municipalities of Yopal and Aguazul in the Department of Casanare. Tick collection was performed at the end of the first dry season, which occurs between December and March. The local landscape is characterized mainly by flat land with very low slopes. The forests are maintained as natural barriers between farms, providing shelter for livestock during the hottest hours of the day and serving as a habitat for a rich population of birds and other wildlife. Ticks were collected from forest sites in protected or semi-preserved native riparian or savannah forests on private farms, close to pastures where cattle and horses grazed, or agricultural activities took place (Fig. 1). Tick collection was performed near water sources (waterfalls, rivers, or ponds), in rural areas located between the coordinates 5°13′21.13"N; 72°30′58.25"W and 5°13′45.23"N; 72°30′58.67"W (Fuya-Oviedo et al. 2022). Ticks were collected from eight forest areas on private farms located close to pastures and with a preserved structure of the original native forest, at altitudes between 186 and 508 m (Fig. 1). The temperature range during the collection period was 15–18 °C.

### Study design, sample size, and sampling strategies

A cross-sectional study design was used to address this study’s objectives. Ticks were collected between 7 a.m. and 3 p.m. over 10 days in March 2021. Low and high dragging techniques (flagging) using a white flannel cloth on the ground and over vegetation (Oliveira et al. 2000) followed by capture via the visual inspection of white clothing (walking trap) were used (Ginsberg & Ewing 1989). Depending on the collection method, larval ticks were kept individually or in clusters. A larval cluster comprises questing larvae that group together at the top of the grass and are collected as a single group in the flannel.

Ticks collected from each location were divided into two groups. The Edge (E) group comprised ticks obtained from the transition area between the grass paddock and the first 30 m of forest, and the Forest (F) group was made up of ticks collected further inside the forest. After collection, all ticks were placed in polypropylene tubes containing RNAlater® (Invitrogen. USA) and transported to the Entomology Laboratory of the National Institute of Health of Colombia in Bogotá for taxonomic and molecular analysis.

### Identification of ticks

Larvae were identified at the genus level using taxonomic keys for ixodid ticks. Nymphs and adult ticks were identified using specific dichotomous keys for ixodid ticks and the genus *Amblyomma* described in Colombia (López-Valencia & Parra-Gil 1985; López-Valencia 2017) and Brazil (Barros-Battesti et al. 2006; Dantas-Torres et al. 2019; Martins et al. 2010; Nava et al. 2014). In parallel, for the nymph groups, four ticks from each collection site were randomly selected for identification via mitochondrial 16S rRNA gene sequencing. In addition, the identity of all ticks that tested positive for the presence of *Rickettsia* DNA was confirmed by sequence analysis of the mitochondrial 16S rRNA gene using previously published primers (Mangold et al. 1998). A Keyence VHX-900F Microscope (Keyence Itasca, IL, USA) was used to take photos at 100x to 200 × magnification.

### DNA extraction

DNA extraction from ticks was performed using a modified Hot-SHOT protocol (Montero-Pau et al. 2008). Briefly, individual ticks (larvae, nymphs, and adults) were placed in tubes and washed three times with 1 ml of phosphate-buffered saline (PBS, pH 7.2), followed by centrifugation at 12,000 × *g*. Adult ticks were cut lengthwise with a sterile blade. One-half of each adult tick was transferred to a tube for DNA extraction and the other half was stored at − 20 °C. Larvae and nymphs were cut into several pieces and processed completely. On the other hand, for the 15 larval clusters, five larvae were taken from each cluster and processed as a single sample. After cutting the ticks, a volume of 50 µL for larvae and nymphs and 90 µL for adults of an alkaline buffer solution (25 mM NaOH, 0.2 mM Na_2_EDTA, pH 12) was added to each tube. The tubes were heated at 90 °C for 30 min and placed on ice for 5 min, after which equal volumes of neutralization solution (40 mM Tris–HCl, pH 5) were added to stabilize the DNA solution. To verify the efficiency of the DNA extraction technique, all samples were subjected to polymerase chain reaction (PCR) amplification of a partial sequence of the 16S mitochondrial rRNA gene using the oligonucleotide primers 16S + 1 5’-CCGGTCTGAACTCAGATCAAGT-3’ and 16S-1 5’-GCTCAATGATTTTTTAAATTGCTG-3’ (Mangold et al. 1998).

### Rickettsia detection

PCR assays for the detection of *Rickettsia* DNA were performed by amplification of a ~ 401 bp fragment of the citrate synthase (*gltA*) gene. As previously described by Labruna et al. (2004), positive samples were subjected to a second PCR to amplify a larger ~ 834 bp fragment of the *gltA* gene (Table 1). In addition, to confirm the classification of these microorganisms as Rickettsiaceae, a 549 bp fragment of the *htrA* gene encoding the 17 kDa common antigen was also amplified in *gltA*-positive samples. Samples testing positive through this PCR strategy include rickettsiae of both the typhus group (TG) and SFG. To confirm the classification of these microorganisms as SFG rickettsiae, a 530 bp region of the outer membrane protein A (*ompA*) gene was amplified in the samples positive for *gltA* and *htrA* (Kato et al. 2013; Regnery et al. 1991). Positive controls for the amplification of mitochondrial 16S rRNA and the *Rickettsia* genes were *Amblyomma mixtum* DNA and *Rickettsia vini* DNA, respectively. The PCR amplification conditions were as described in Table 1. *Rickettsia* species identification was performed by *gltA* gene sequencing and subsequent comparison with GenBank entries via a BLASTn search.

**Table 1:** Primer sets used for detection and identification of Tick and *Rickettsia* species

Primers	Gen	Nucleotide sequences (5’−3’)	Approximate amplicon size (bp)	Reference
CS239 F	*glt*A	GCTCTTCTCATCCTATGGCTATTAT	834	Labruna et al. (2004)
CS1069 R		CAGGGTCTTCGTGCATTTCTT		
CS-78		GCAAGTATCGGTGAGGATGTAAT	401	
CS-323		GCTTCCTTAAAATTCAATAAATCAGGAT		
Rr190.70p	*ompA*	ATGGCGAATATTTCTCCAAAA	530	Labruna, et al. (﻿^2004^) Regnery et al. (1991)
Rr190.602n		AGTGCAGCATTCGCTCCCCCT		
17 k-5	*htrA*	GCTTTACAAAATTCTAAAAACCATATA	549	
17 k-3		TGTCTATCAATTCACAACTTGCC		

### Electrophoresis and analysis of results

The PCR products (10 µL) were loaded onto a 1% agarose gel (UltraPureTM LMP Agarose, Invitrogen®, USA) and separated by electrophoresis (5 V/cm). Gels were stained with RedSafe® DNA Stain, BulldogBio, New Hampshire, USA (1µL/mL) and visualized in a UV light transilluminator (ChemiDoc XRS + System, Bio-Rad, USA). The size of the amplified fragments was estimated by comparison with a 100 bp ladder (GeneRuler 100 bp DNA Ladder, Thermo Scientific, USA).

### Sequencing

DNA was cleaned directly from the PCR reactions using the DNA Clean & Concentrator-5 Kit (Zymo Research Corporation, Irvine, CA, USA) according to the manufacturer’s instructions. The purified products were Sanger-sequenced by LGC Genomics (Berlin, Germany). The resulting sequences were aligned, and quality trimming was performed to remove poor 3’ and 5’ end reads using the Unipro Ugene® program. The sequences were then compared to other sequences deposited in GenBank using BLASTn.

### Sequence alignment and phylogenetic tree construction 

Phylogenetic inference from the *gltA* and *htrA* sequence data generated during this study was based on the maximum-likelihood criterion. To ascertain the relationships with known *Rickettsia gltA* and *htrA* sequences, the dataset for phylogenetic inference additionally comprised published sequences retrieved from GenBank. Since models of nucleotide substitution can bias the accuracy of the phylogenetic inference, both likelihood scores and estimated model parameters were used to rank a candidate set of nucleotide substitution models using the Akaike Information Criterion. Akaike weights were used as evidence to identify the most suitable model given the data. The model evaluation steps and maximum-likelihood tree search algorithms were implemented in jModelTest 2.1.10 (Posada 2008) and PAUP 4.0, beta version (Swofford 2002; Wilgenbusch & Swofford 2003), respectively. Branch support was calculated using 1000 bootstrap replicates.

## Results

In total 852 individual ticks were collected. Of these, 230 (26.9%) were larvae, 488 (57.3%) were nymphs, and 134 (15.7%) were adults (71 females and 63 males). In addition, 15 groups of larval clusters were collected. For subsequent procedures, 5 larvae from each cluster were pooled and processed as a single sample, giving a total of 15 additional tick samples.

**Fig. 2:**
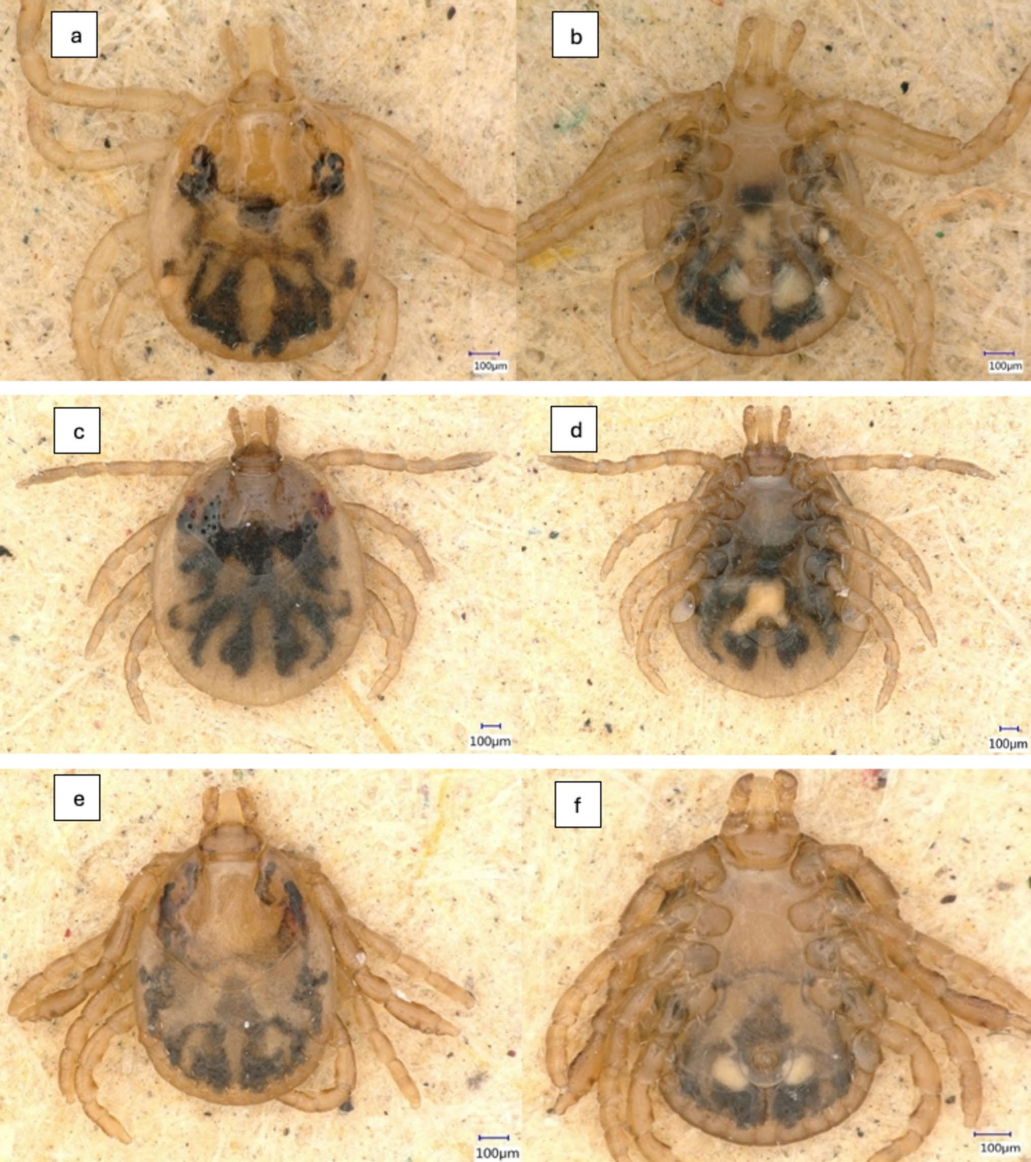
Dorsal and ventral views of nymphs of (**a**, **b**) *Amblyomma mixtum*, (**c**, **d**) *Amblyomma dissimile*, (**e**, **f**) *Amblyomma* cf. *parvum*

Based on morphological identification, 98.3% (226/230) of the larvae were classified as *Amblyomma* spp. and 1.7% (4/230) as *Dermacentor* spp. Of these larvae, those that later tested positive for *Rickettsia* spp. infection were species-confirmed by sequencing the mitochondrial 16S rRNA gene. Thus, 13 larvae were identified as *Amblyomma dissimile* and three as *Amblyomma* cf. *parvum* (GenBank accession nos. ON679575 to ON679587 and ON680753 to ON680755, respectively). Regarding the nymphs, 99.6% (486/488) were initially identified as *Amblyomma cajennense* sensu lato (s.l.), 0.2% (1/488) as *A. dissimile*, and the remaining 0.2% (1/488) as *A.* cf. *parvum* (Fig. 2). Analysis of the 16S rRNA mitochondrial gene sequences of the 32 nymphs selected from the eight collection sites (four nymphs/collection site) previously identified as *A. cajennense* s.l. showed that they were *A. mixtum* (GenBank accession nos. ON680714 to ON680746). The 486 nymphs classified as *A. cajennense* s.l. were therefore inferred to be *A. mixtum*. Nymphs of *A. dissimile* and *A*. cf. *parvum* were also confirmed by sequencing a region of the 16S mitochondrial DNA (GenBank accession nos. ON679574 and ON680752, respectively). All adult ticks were identified as *A. mixtum*. The distribution and abundance of *A. mixtum* ticks varied among the study sites (Table 2).

**Table 2:** Number of ticks by species and stage according to their distribution in the forest areas or fragments (F#) of the municipalities of Yopal and Aguazul, Casanare.** B**: border of the forest segment, **I**: internal area of the forest

Stage	Species	F1		F2		F3		F4		F5		F6		F7		F8		Total
		B	I	B	I	B	I	B	I	B	I	B	I	B	I	B	I	
Larvae	*Amblyomma* spp*.*	12	0	2	26	4	52	5	28	0	22	4	28	10	17	3	13	**226**
	*Dermacentor* spp*.*	0	0	0	0	4	0	0	0	0	0	0	0	0	0	0	0	**4**
Nymphs	Amblyomma mixtum*^s^	3	10	28	46	10	32	0	68	5	17	17	11	50	73	60	56	**486**
	Amblyomma dissimile**	0	0	0	0	0	0	0	1	0	0	0	0	0	0	0	0	**1**
	*Amblyomma* cf. parvum**	0	0	0	0	0	0	0	0	0	0	0	1	0	0	0	0	**1**
Adults	*Amblyomma mixtum*	0	7	1	18	5	19	0	1	2	9	11	9	0	20	2	30	**134**
Total ticks by fragment's area	Total ticks by fragment	15	17	31	90	23	103	5	98	7	48	32	49	60	110	65	99	**852**
Total ticks		**32**		**121**		**126**		**103**		**55**		**81**		**170**		**164**		**852**
Larvae clusters	*Amblyomma* spp*.*	0	5	0	1	0	5	0	0	0	2	0	1	0	0	0	1	**15**

**Classified by 16S mitochondrial DNA amplification and sequencing *^S^ After taxonomic classification using morphological identification keys, 5 ticks from each collection fragment (F#) were species confirmed by PCR and 16S mitochondrial DNA sequencing

**Fig. 3:**
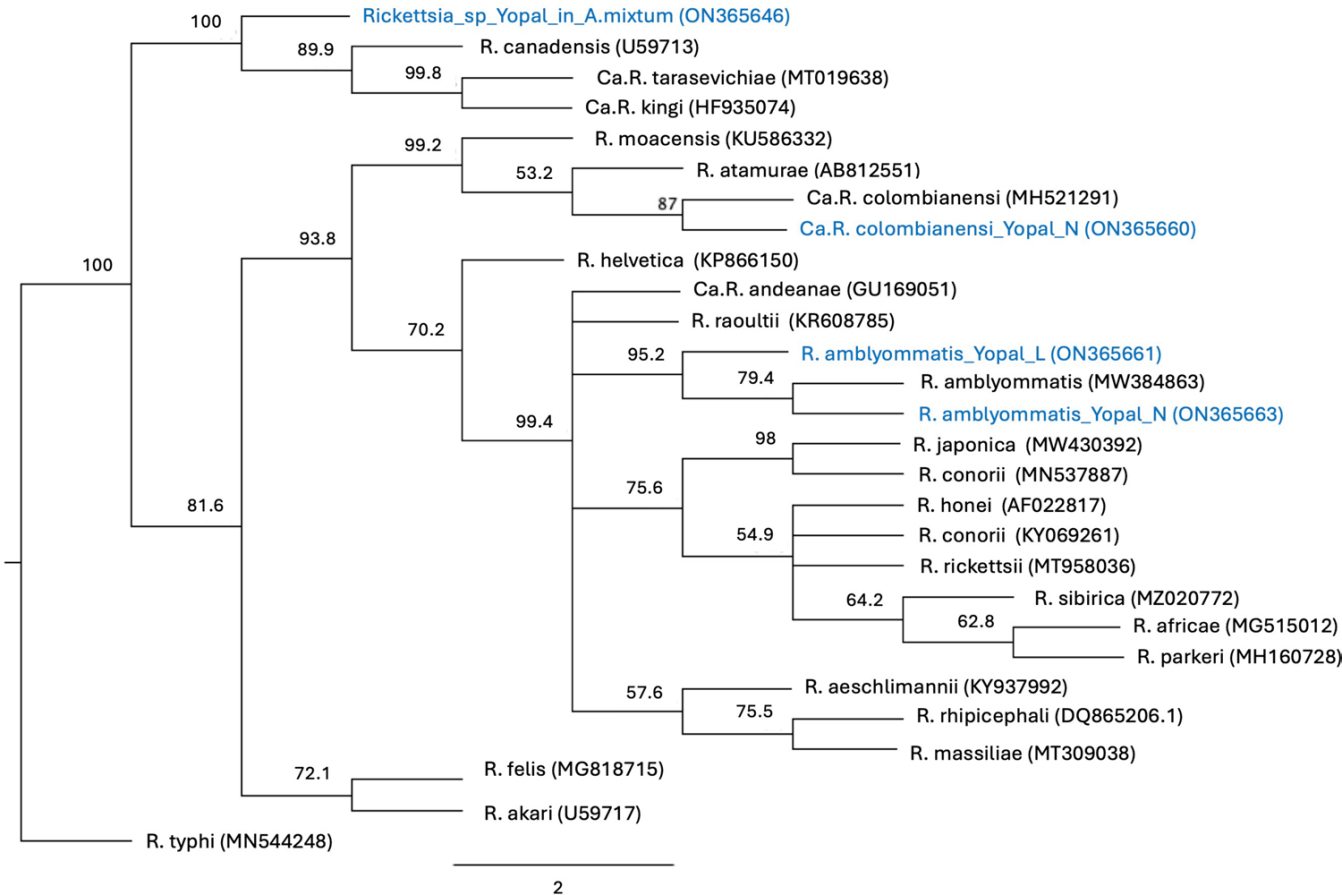
Maximum-likelihood tree constructed from *gltA* partial sequences (834 bp) for *Rickettsia* sp. Numbers represent bootstrap support generated from 1000 replications. GenBank accession numbers are in brackets

*Rickettsia* sp. was detected in 2.2% (19/852) of the tick samples; 0.35% (3/852) were found in forest edge and 1.85% (16/852) in forest. Positive samples were found in four out of the eight forest sites visited. Amplification of the *ompA* gene showed that 7.8% (16/230) of the larvae, 0.2% (1/488) of the nymphs, and 1.5% (2/134) of the adult ticks belonged to the SFG rickettsiae. Sequences of the *gltA* gene from these 19 DNA samples revealed the presence of three different *Rickettsia* species as follows: 13 *gltA* sequences from larvae were 100% identical to “*Candidatus* Rickettsia colombianensi” (GenBank accession nos. ON365648 to ON365660), and two DNA samples from adult male ticks showed the presence of a *Rickettsia* sp. (GenBank accession nos. ON365646 and ON365647) with 99.6% identity to an endosymbiotic *Rickettsia* of *Amblyomma tonelliae,* named “*Rickettsia* sp. strain El Tunal” (GenBank accession no. KP171629), identified in Argentina (Tarragona et al. 2015) (Table 3). Phylogenetically, this rickettsia is closely related to *Rickettsia monteiroi* (95.2% identity with the *R. mointeroi* species, GenBank accession no. FJ269035; Fig. 3).

**Table 3:** *Rickettsia* species found in the different stages of the collected ticks

Total ticks collected	Stage	Tick species	N* 430 bp	*Rickettsia* species identified by sequencing 850 bp *gltA* to 430 bp gltA-positive samples (Number of positive ticks) [Tick species]
852 individual ticks	Adults	*Amblyomma mixtum*	134	*Rickettsia sp. Yopal genotype of A. mixtum* (2)
	Nymphs	*Amblyomma cajennense* s.l	486	0
		*Amblyomma dissimile*	1	0
		*Amblyomma cf. parvum*	1	*R. amblyommatis* (1)
	larvae	*Amblyomma* spp*.*	226	*Ca*. Rickettsia colombianensi (*N* = 13)
				[*Amblyomma dissimile*]
				*R. amblyommatis* (*N* = 3)
				[*Amblyomma* cf. *parvum*]
		*Dermacentor* spp.	4	0
Larvae in clusters	Larvae	*Amblyomma* spp*.*	15	0

N* 430 bp Number of ticks with a 430 bp fragment amplified

The *gltA* sequences found in three larvae and one nymph of *A.* cf*. parvum* ticks (GenBank accession nos. ON365661 to ON365663) had 100% identity with GenBank accession nos. CP015012.1, CPO12420, and MW539676 of *Rickettsia amblyommatis* (Table 3). Amplification of this gene from *Dermacentor* spp. was not detected.

**Fig. 4:**
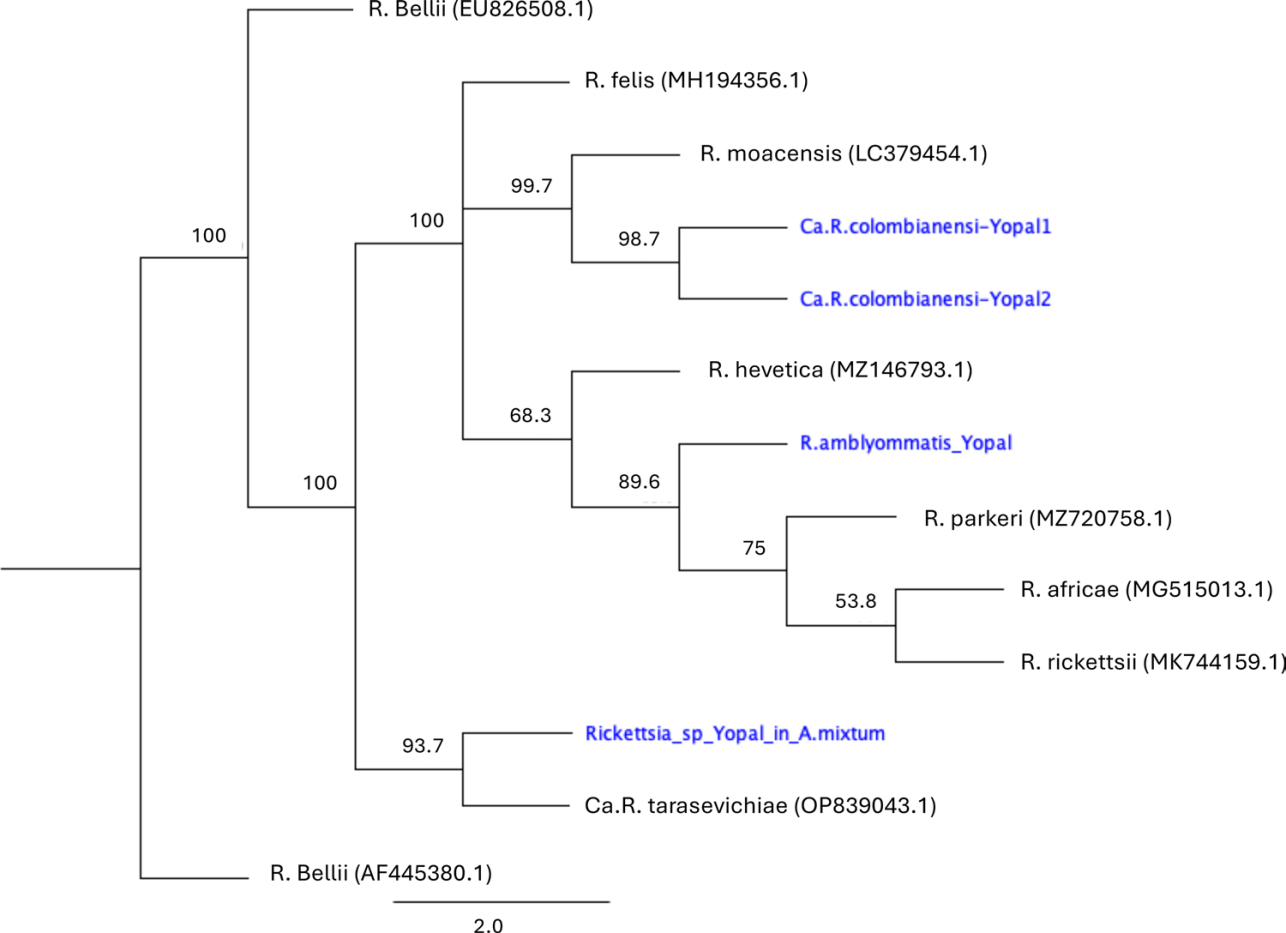
Maximum-likelihood tree constructed from *htrA* partial sequences (549 bp) for *Rickettsia* sp. Numbers represent bootstrap support generated from 1000 replications. GenBank accession numbers are in brackets

The *htrA* partial sequences from samples corresponding to “*Ca*. R. colombianensi” rickettsiae (GenBank accession nos. PP253961 and PP253962) were 98.97% identical to the corresponding sequence of *Rickettsia monacensis* (GenBank accession no. LC379454). The *htrA* sequence (GenBank accession no. PP274031) was 100% identical to *R. amblyommatis* strain Ac37 from *A. cajennese* (GenBank accession no. CP012420), and the sequence from *Rickettsia* sp. Yopal in *A. mixtum* (GenBank accession no. PP274032) was 100% identical to “*Rickettsia* sp. strain El Tunal” in *A. tonelliae* (GenBank accession no. KP171630) and shared a high identity (89.94%) with “*Candidatus* Rickettsia tarasevichiae” (GenBank accession no. OP839043; Fig. 4).

## Discussion

Specimens of all three tick stages (larvae, nymphs, and adults) were found at all collection sites. *A. mixtum* nymphs were the most abundant tick species collected. *A. mixtum* is a tick species belonging to the *A. cajennense* s.l. complex and one of the 29 known *Amblyomma* species described in Colombia (Acevedo-Gutiérrez et al. 2020). This is consistent with the documented behavior of *A. mixtum*, where the three stages use different habitats during the dry season, with a preference for riparian areas. Moreover, of the three stages, adults were the fewest in number, which may be because, during this time of the year, adult *A. mixtum* ticks have a greater preference for pasture areas compared to forest areas (Forero-Becerra et al. 2022).

To our knowledge, few records of *A*. cf. *parvum* in Colombia have been published. This study represents the fourth documented record of *A*. cf. *parvum* in Colombia. Infestations in armadillos (*Dasypus sabanicola*) (Doss, 1978), dogs (*Canis familiaris*) (López-Valencia & Parra-Gil 1985), and equines (Santodomingo et al. 2019) have been reported. Both armadillo burrows and equines were frequently observed in the collection areas.

On the other hand, the presence of *A. dissimile* is associated with the presence of reptiles and amphibians that are common in the riparian forests of the Colombian Orinoquia.

As documented in previous studies, *Ca*. R. colombianensi has been found in *A. dissimile* ticks. *Ca*. R. colombianensi is a *Rickettsia* incertae sedis of the SFG rickettsiae and is widely distributed throughout the American continent. The presence of this *Rickettsia* has also been reported in Honduras (Novakova et al. 2015), Costa Rica (Moreira et al. 2015), Brazil (Luz et al. 2018; Ogrzewalska et al. 2019), Mexico (Sánchez-Montes et al. 2019), and El Salvador (Mateus-Anzola et al. 2020).

Moreover*, Ca.* R. colombianensi has been described in various regions of Colombia (Cárdenas 2020; Cardona et al. 2020; Contreras-Ortega 2014; A. Cotes-Perdomo et al. 2018, 2022; Londoño et al. 2014; Martínez-Sánchez et al. 2021; Miranda et al. 2012, 2020; Sánchez et al. 2019)*.* It has also been found to infect ticks of the species *Rhipicephalus (Boophilus) microplus*, *A. cajennense* s.l., *Amblyomma patinoi*, and *Rhipicephalus sanguineus* s.l. (Miranda et al. 2012; Rodríguez-Bautista 2022).

*Ca.* R. colombianensi has a high genetic similarity with *R. monacensis*, *Rickettsia heilongjiangensis,* and *Rickettsia tamurae* (Fig. 3). This finding suggests that *Ca*. R. colombianensi is closely related to the SFG rickettsiae and could constitute a public health concern. Additional research is required to elucidate its epidemiological significance for both animal and human public health.

The novel *Rickettsia* species was named “*Rickettsi*a sp. Yopal genotype of *A. mixtum.*” It is phylogenetically similar to the *canadensis* group of *Rickettsia*. In particular, it is closely related to *Rickettsia canadensis*, *R. monteiroi*, and “*Ca.* R. tarasevichiae” (Fig. 3). The *gltA* sequences of the *Rickettsia* Yopal genotype of *A*. *mixtum* differed by a maximum of 5.5% from the *gltA* sequences of *R. monteiroi, R. canadensis,* “*Candidatus* Rickettsia kingi”, and “*Ca*. R. tarasevichiae” (GenBank accession nos. FJ269035, CP003304, HF935074, and KM288462, respectively).

Given its phylogenetic relationship with other non-pathogenic rickettsiae (Parola et al. 2013; Tarragona et al. 2015), it could be suggested that this microorganism is non-pathogenic to humans. However, such assumptions should be made with caution, as another member of this group, “*Ca*. R. tarasevichiae” has been associated with human fatalities in China (Jia et al. 2013; Liu et al. 2016) and Russia (Rudakov et al. 2019).

It is interesting to note the close phylogenetic relationship of the new “*Rickettsia* Yopal genotype in *A. mixtum*” with *R. monteiroi*, *R. canadensis*, “*Ca*. R. tarasevichiae” and *Rickettsia* sp. strain El Tunal from Argentina despite their distant geographical locations and the phylogenetically distant tick species in which they were found. *R. monteiroi* was associated with *Ambyomma incisum* (Pacheco et al. 2011), *R. canadensis* with *Haemaphysalis leporispalustris* (McKiel et al. 1967; Parola et al. 2013) and *Haemaphysalis japonica* (Igolkina et al. 2018) and “*Ca*. R. tarasevichiae” with *Ixodes persulcatus* (Eremeeva et al. 2006), *Haemaphysalis japonica douglasii* (Mediannikov et al. 2006), and *Dermacentor silvarum* (Igolkina et al. 2018).

Although this novel *Rickettsia* appears to belong to the *canadensis* group, which includes non-fatal SFG rickettsiae (Fournier et al. 2006; Merhej & Raoult 2011; Parola et al. 2013), complementary studies such as the characterization of other loci, isolation of the microorganism, and identification of possible hosts and/or reservoirs are needed to support the epidemiological features of a possible association with human rickettsiosis.

Although *R. amblyommatis* is likely one of the most abundant and widespread SFG rickettsiae species in the Americas, with infection rates exceeding 40% in *Amblyomma americanum* ticks in the United States (Karpathy et al. 2016), it was detected at a very low frequency in the tick population in this study. This *Rickettsia* species was found exclusively in *A*. cf. *parvum*, making this study the first report of *R. amblyommatis* infecting *A.* cf*. parvum* in Colombia.

Although *R. amblyommatis* has not been confirmed as a human pathogen, serological evidence suggests that humans develop an immune response to this organism and that it may be associated with disease manifestations in some patients (Apperson et al. 2008).

Although *Ca*. R. colombianensi and *R. amblyommatis* belong to the SPF Rickettsiae, their pathogenicity is unknown and their medical relevance should be considered with caution. Despite major advances in the study of these specific microorganisms and other tick-associated pathogens in Colombia, very little has been reported on the potential role of wildlife and their ticks in the epidemiology of human and animal diseases.

## Conclusions

Ticks of the species *A. dissimile*, *A*. cf. *parvum*, and *A. mixtum* were identified in forest fragments in rural areas of the municipalities of Yopal and Aguazul. Rickettsiae of the species “*Ca*. R. colombianensi” and *R. amblyommatis* were found infecting *A. dissimile* and *A*. cf. *parvum* ticks, respectively. In addition, a new *Rickettsia* species, named “*Rickettsia* sp. Yopal genotype of *A. mixtum*,” was discovered in a forested area; this is the first report of a *Rickettsia* species belonging to the canadensis group in Colombia.

## Supplementary Information

Below is the link to the electronic supplementary material.

Supplementary file1 (PDF 2135 KB)

## Data Availability

Information on coordinates and the inventory of tick collections can be consulted in the open access area of the SiB Colombia website https://ipt.biodiversidad.co/sib/resource?r=ins_garrapatas (Spanish version only). DNA sequences can be found in the Nucleotide Database on the National Center for Biotechnology Information (NCBI) website https://www.ncbi.nlm.nih.gov/nucleotide/ searching for the accession number.

## Declarations

### Consents

Consent to participate and consent to publish are not applicable.
