# Supplementary material for: Detection of Rickettsia spp. in questing ticks (Acari: Ixodidae) from forest fragments adjacent to agricultural and livestock farms in Casanare, Colombia
Source: Parasitol Res. 2025 Apr 24;124(4):44. doi: 10.1007/s00436-025-08484-2 (PMC12021727; doi:10.1007/s00436-025-08484-2)

Supplementary file of the manuscript:

Detection of *Rickettsia* spp. in questing ticks (Acari: Ixodidae) from forest fragments adjacent to agricultural and livestock farms in Casanare, Colombia

Next files correspond only to the most representative samples (positive DNA samples only) gels made for the different genes amplified (16S mRNA for tick species identification and *gltA*, *htrA* and *ompA* for Rickettsia detection and identification).

The total number of gels in this study was over 90 gels.

I hope this completes the information required by the reviewers. In any case, I remain attentive to any other request.

16*S* mitochondrial *RNA gene amplification* for tick species identification

- The gel in the next slide shows the nymph DNA samples that were amplified for the 16*S mRNA* gene.
- As mentioned in the manuscript, only samples from *Rickettsia* sp. positive adult ticks or larvae were amplified for the 16S mRNA gene. Gels are not shown.

16S *mRNA* amplification. Nymphs DNA.

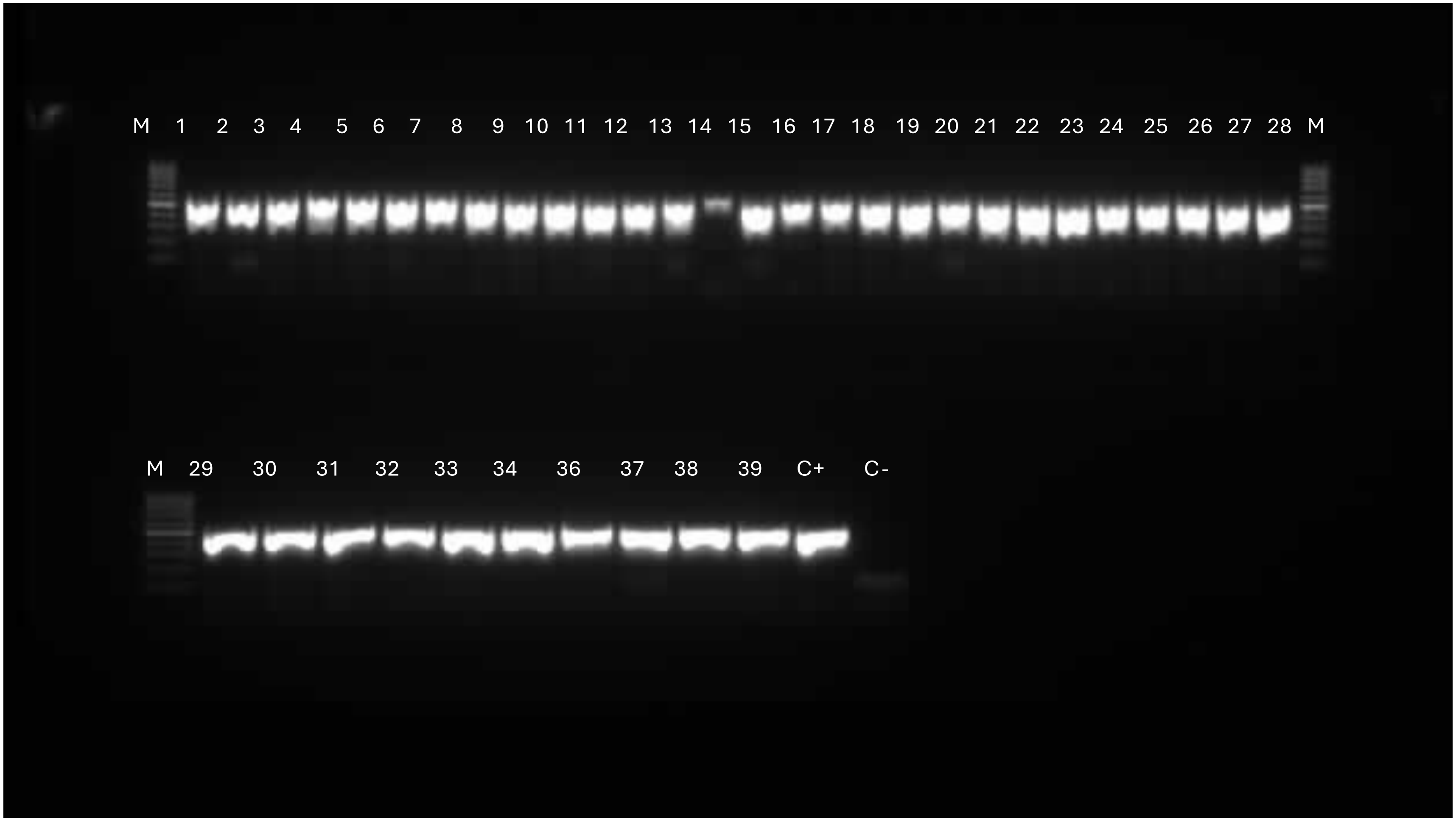

gltA gene amplification

- The following slides are related to the amplification of the *gltA* gene (400bp or 800bp fragment size) in DNA samples from larval, nymph and adult ticks. Only a few are shown. As positive results were obtained, they were sent for sequencing.
- *800bp size fragments were used for sequencing

Adults plate1
(*A. cajennense*)
Gel 3. ***gltA*** gene

gltA

Result:
1 positive sample
#36 (*)

400bp

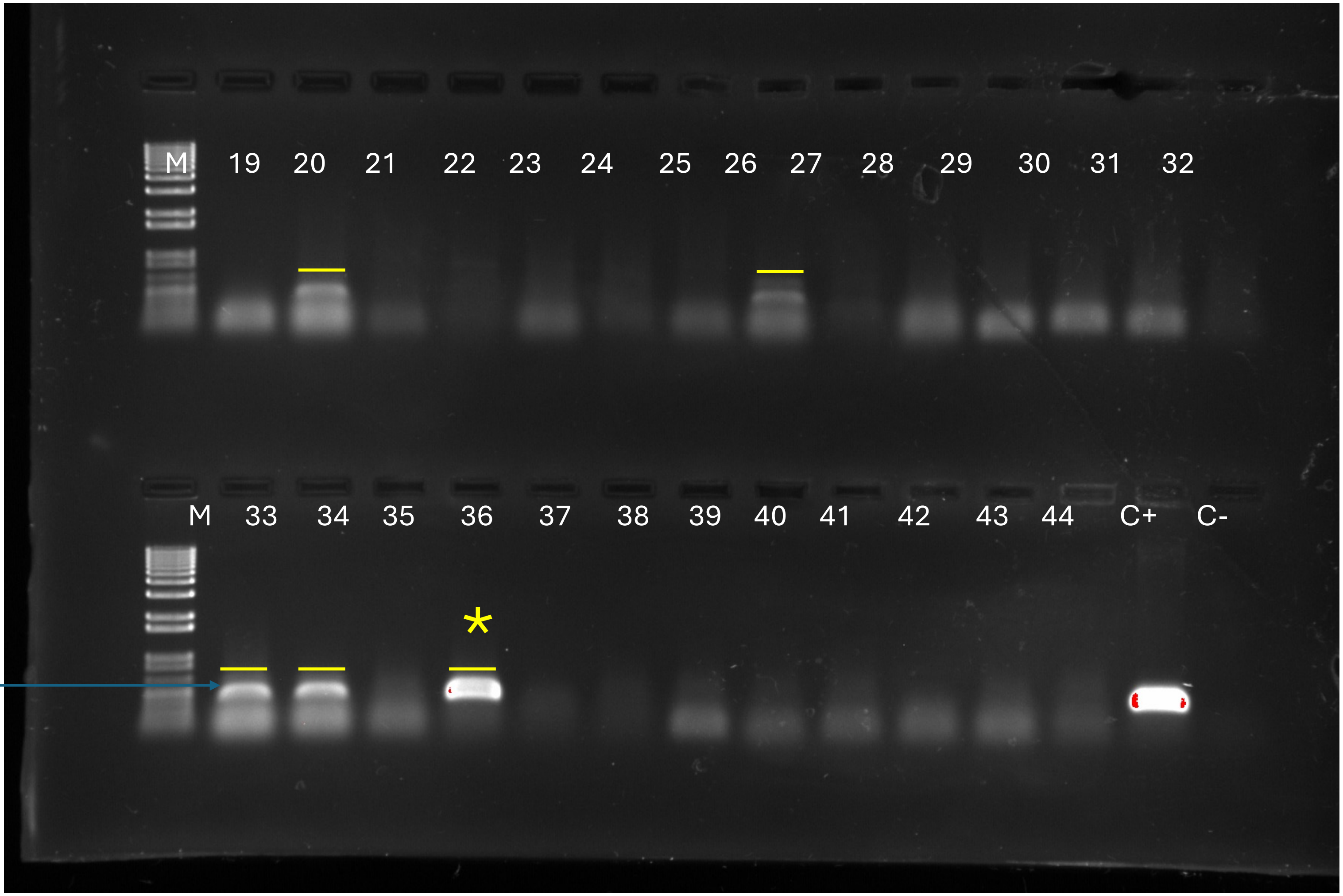

gltA 400. Adult ticks Yopal and Nocaima Diluted DNA 1:10

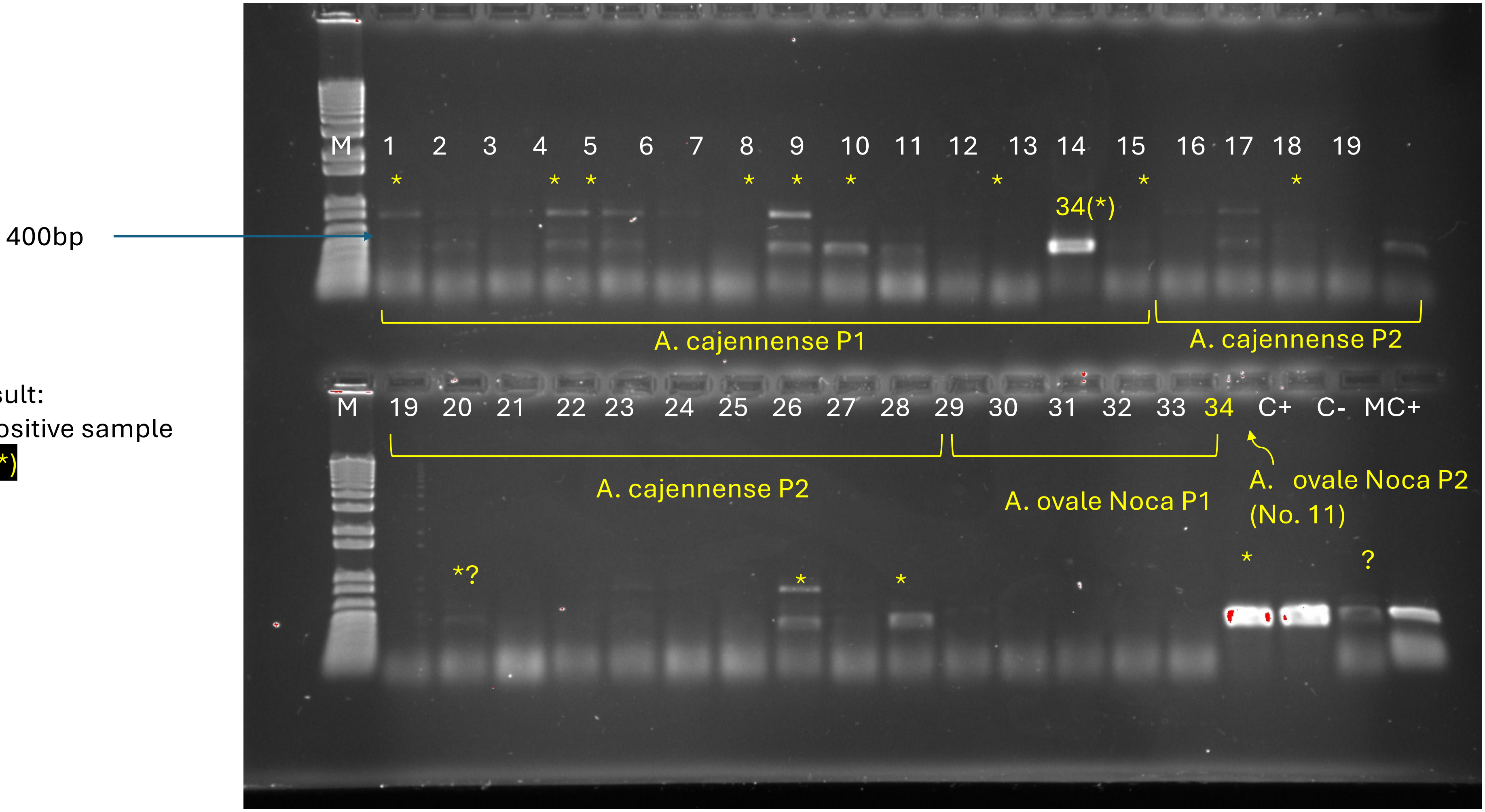

Result:
1 positive sample
37(*)

gltA (800bp). **Larvae**. Plate 1

800bp

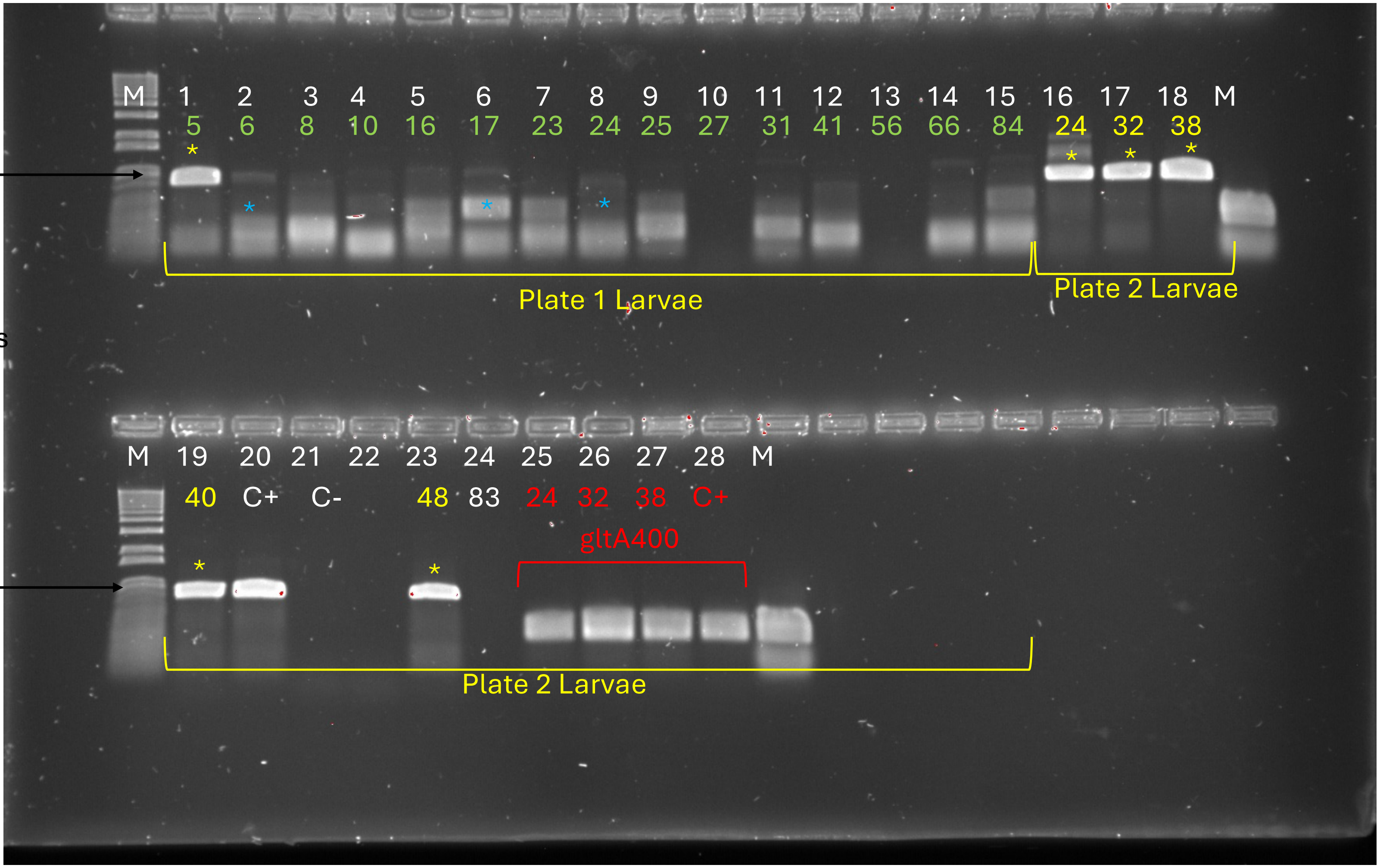

800bp

Result:
6 positive simples
(*)

gltA Confirmative. Gel 1. Samples: Positive gltA 400bp) from plate **2 Larvae** (N:4), Plate 3 larvae (N:15), Plate 1 Adults (N:8) y plate 2 Adults (9).

400bp

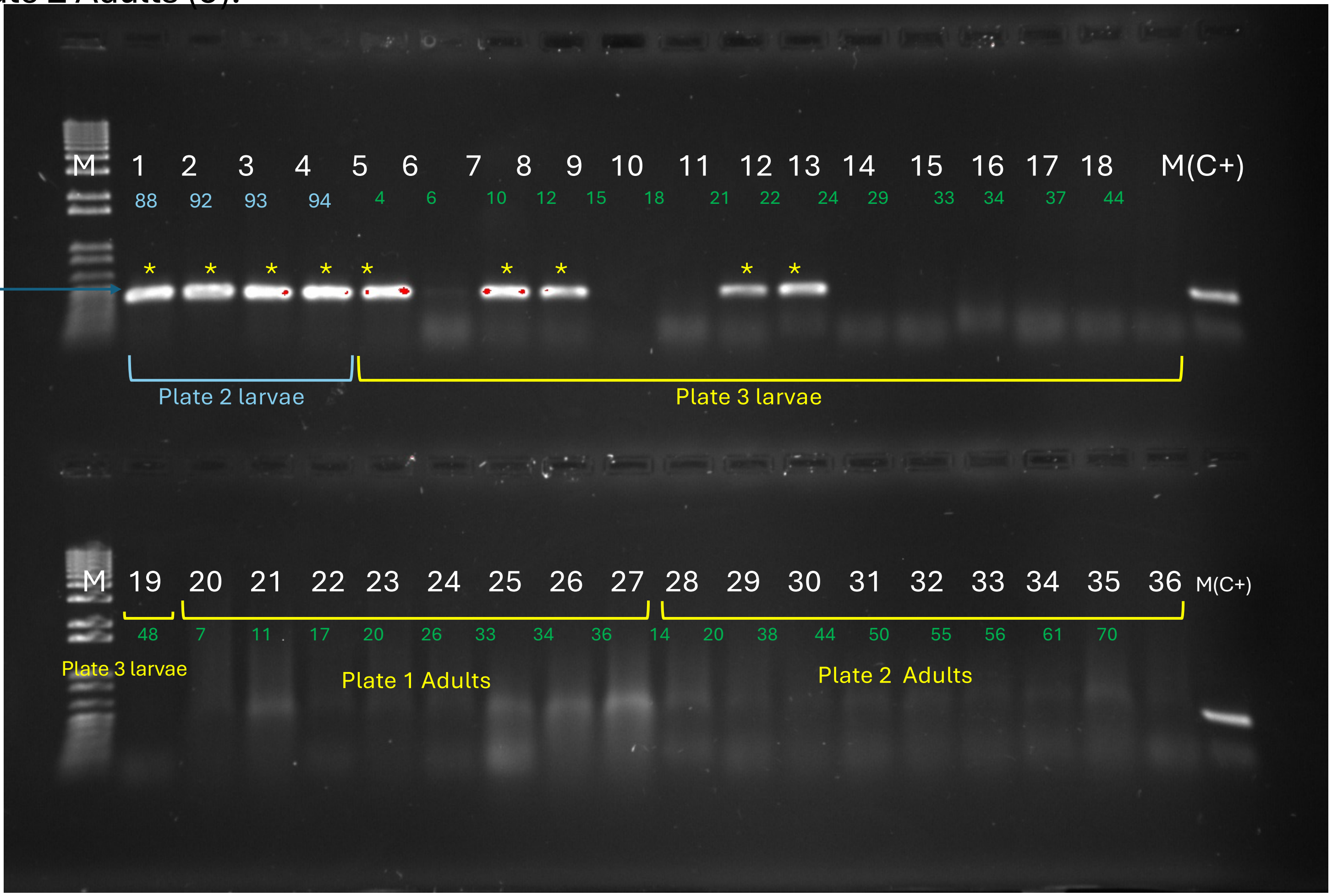

Result:
9 positive sample
37(*)

gltA 800. **Larvae** - Plate 1, 2 y 3 & Nymphs plates 1, 2 y 3 . Gel 1

Larvae

gltA

800bp

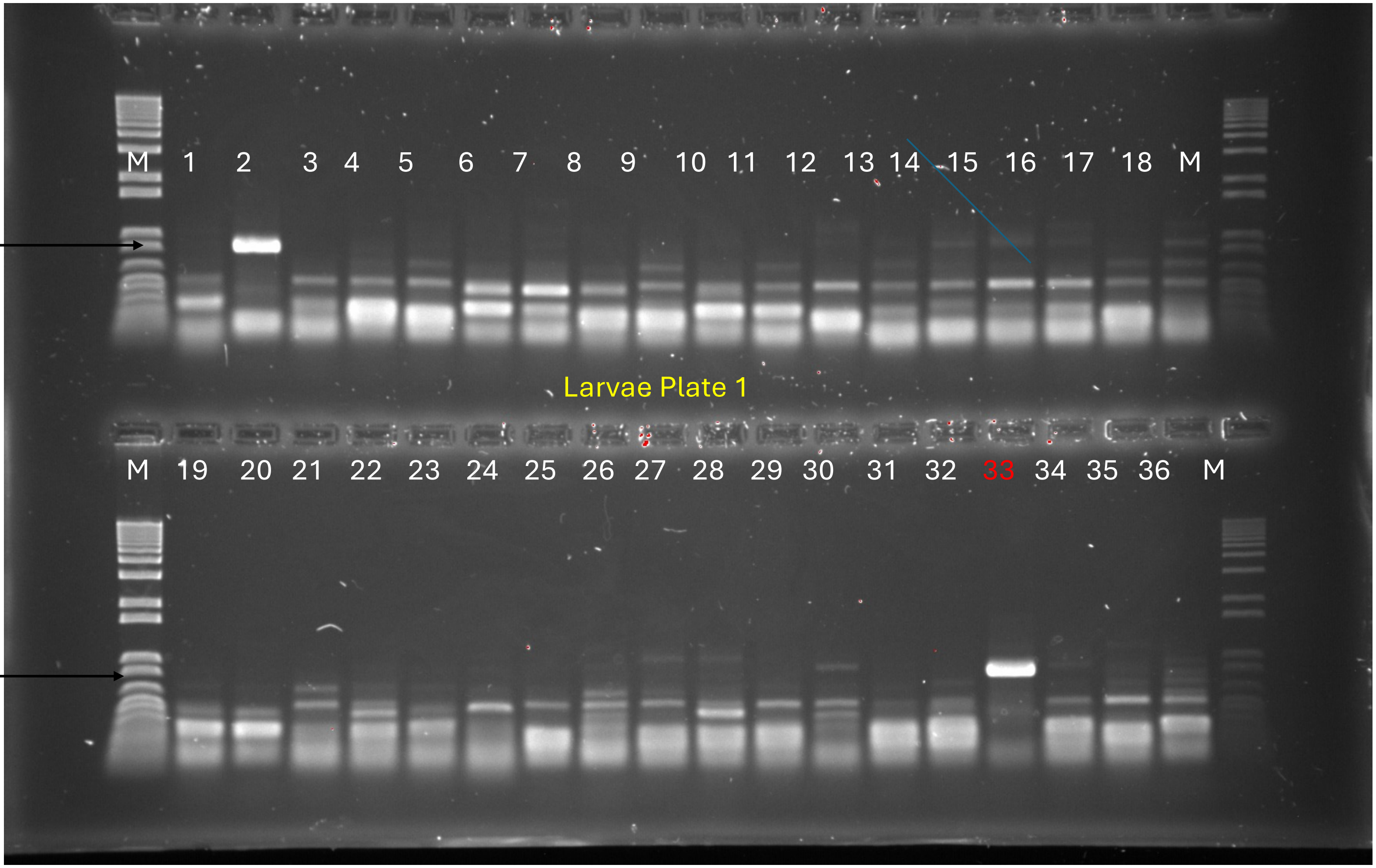

800bp

Result:
2 positive sample
37(*)

800bp

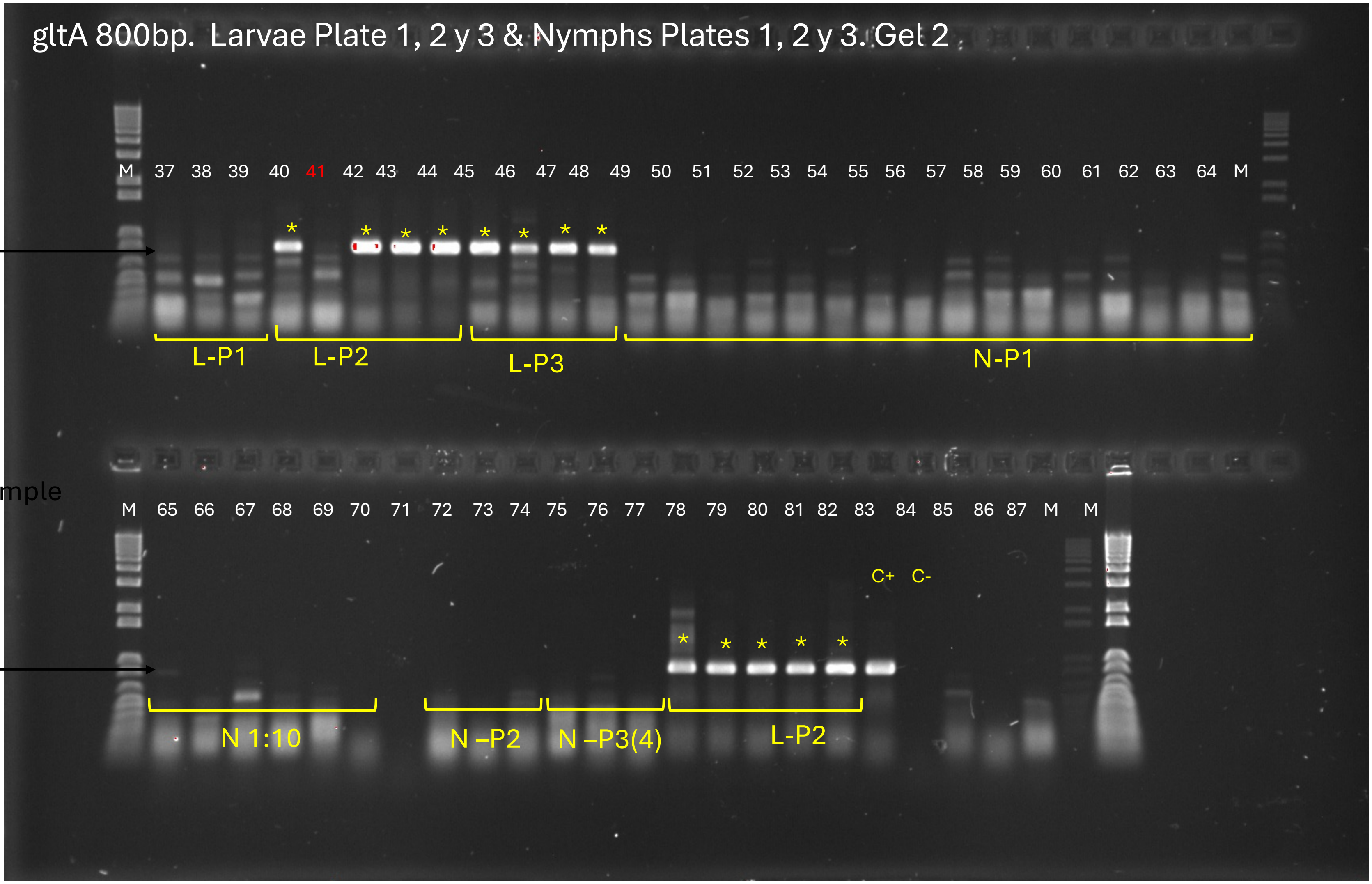

Result:
13 positive sample
(*)

800bp

htrA gene amplification

The following slide shows the amplification of *htrA* gene fragments from larval DNA samples that were positive for the *gltA* gene.

htrA gene.
Larvae DNA

549bp

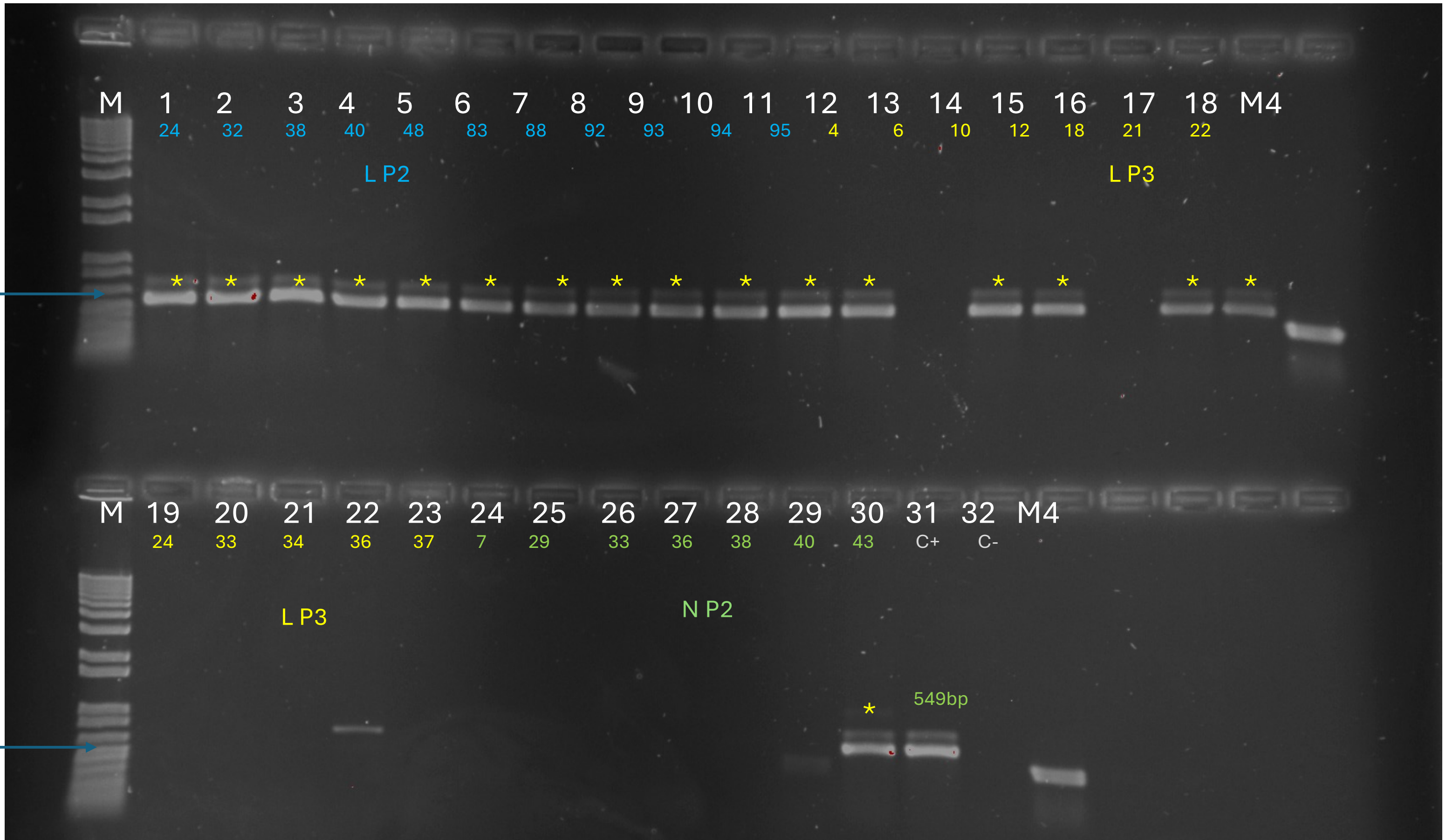

htrA gene. Positive larvae to gltA 400bp & gltA 800bp of plates 2 & 3, and larvae 5, 36 y 55 of plate 1 (10ul PCR product/well).

549bp

Result:
16 positive simples from larvae
1 positive sample from Nymph
(*)

ompA amplification

- The following slide shows the amplification of *ompA* gene fragments from some larval DNA samples that were positive for the *gltA* gene.

ompA PCR. Larvae Plate 1 (N:49). 7 September 2021

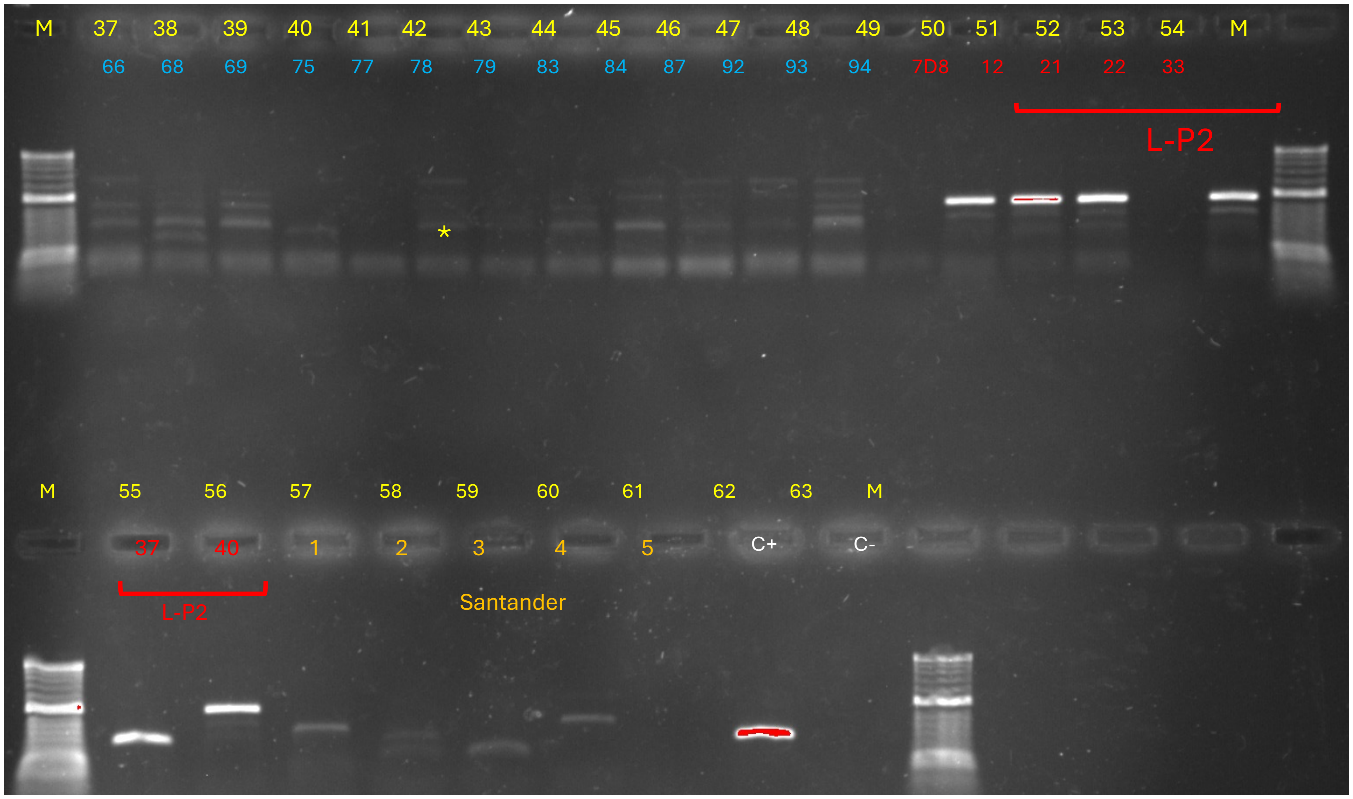

Supplement: Supplementary file 1 — Supplementary file1 (PDF 2135 KB) [file 436_2025_8484_MOESM1_ESM.pdf]
